# Association of Subclinical Hypothyroidism with Dyslipidemia and Increased Carotid Intima-Media Thickness in Children

**DOI:** 10.4274/jcrpe.3719

**Published:** 2017-06-01

**Authors:** Edip Unal, Alper Akın, Ruken Yıldırım, Vasfiye Demir, İsmail Yildiz, Yusuf Kenan Haspolat

**Affiliations:** 1 Dicle University Faculty of Medicine, Department of Pediatric Endocrinology, Diyarbakır, Turkey; 2 Dicle University Faculty of Medicine, Department of Pediatric Cardiology, Diyarbakır, Turkey; 3 Dicle University Faculty of Medicine, Department of Family Medicine, Diyarbakır, Turkey; 4 Dicle University Faculty of Medicine, Department of Biostatistics, Diyarbakır, Turkey

**Keywords:** Subclinical hypothyroidism, carotid intima-media thickness, dyslipidemia, childhood

## Abstract

**Objective:**

Subclinical hypothyroidism (SH) is defined as an elevated serum thyroid-stimulating hormone (TSH) level with free thyroxine (fT_4_) level in the normal range. There are very few studies in the literature reporting on the effect of SH on lipid metabolism and carotid intima-media thickness (CIMT) in children.

**Methods:**

The study included 38 children diagnosed with SH and a control group comprising 38 healthy, euthyroid children. SH was diagnosed based on an elevated TSH level (4.2-20 mIU/L) and normal fT_4_ level measured in two morning fasting blood samples obtained at an interval of 2 to 6 weeks. Blood samples were collected by venipuncture in the morning after an overnight fast.

**Results:**

The patient group included 38 children (16 male, 22 female) with SH and the control group -38 healthy, euthyroid children (20 male, 18 female). Mean age was 8.1±3.6 (range, 3.5-15) years in the patient group and 8.9±2.4 (range, 4.5-15) years in the control group. In the patient group, total cholesterol (TC), low-density lipoprotein cholesterol (LDL-C), TC/high-density lipoprotein cholesterol (HDL-C), and LDL-C/HDL-C were higher compared to the control group (p=0.049, p=0.014, p=0.002, and 0.003, respectively). In the patient group, CIMT was also significantly higher compared to the control group (p=0.001). The patient group was further divided into two subgroups based on their serum TSH level: (I) patients with mildly elevated TSH (TSH=4.2±10 mIU/L) (n=33) and (II) patients with high TSH (TSH≥10 mIU/L) (n=5). However, no significant difference was found between the patients with mild and severe SH with regard to TC, LDL-C, HDL-C, triglyceride level and CIMT levels (p=0.635, p=0.424, p=0.310, p=0.342, and 0.610, respectively).

**Conclusion:**

Subclinical hypothyroidism leads to increased dyslipidemia (increased TC and LDL) and increased CIMT, which leads to increased risk of cardiovascular disease. Further studies are needed to substantiate these findings in children with SH.

## What is already known on this topic?

Subclinical hypothyroidism (SH) increases the intima-media thickness (CIMT) of the carotid artery. SH in children is also associated with increased total cholesterol (TC) and low-density lipoprotein cholesterol (LDL-C) levels as well as increased TC/high-density lipoprotein (HDL) and LDL/HDL ratios.

## What this study adds?

Our study is of prime value since there are very few studies reporting on lipid profile abnormalities and increased CIMT in children with SH, as opposed to a large number of studies in adults. The present study is the first study in the literature to show increased TC and LDL-C as well as increased TC/HDL and LDL/HDL ratios in non-obese children with SH.

## INTRODUCTION

Subclinical hypothyroidism (SH) is defined as a state of elevated serum thyroid-stimulating hormone (TSH) level with a free thyroxine (fT_4_) level in the normal range (1). Depending on the level of serum TSH, SH is divided into mild (TSH=4.2-9.9 mIU/L) and severe (TSH≥10 mIU/L). Mild SH constitutes almost 75% of the patients with SH (2). SH affects 3-18% of the adult population and this prevalence increases with age (3). The prevalence of SH in children is reported to range between 1.7-9.5% (4,5). The most common cause of SH in children, as in adults, is Hashimoto’s thyroiditis (4). There is no consensus on an ideal treatment for the management of SH. Moreover, whether dyslipidemia and increased carotid intima-media thickness (CIMT) in SH should be treated remains controversial.

Available data from adult studies and from few pediatric studies indicate that SH is associated with an alteration in lipid profile (3,6,7). In addition, SH has also been shown to have impact on carbohydrate metabolism, the neuromuscular system, and on cognitive functions (8,9). However, SH has its major effects on the cardiovascular system. Atherosclerosis is an important factor affecting the incidence of cardiovascular disease. Although there is no consensus on the association between SH and atherosclerosis, atherosclerosis is considered to be triggered by subintimal lipoprotein deposition and endothelial dysfunction. CIMT is used as a marker of atherosclerosis (2). Literature reviews show that the studies reporting on the effect of SH on lipid metabolism and CIMT have been mainly conducted in adults and that studies in children are sparse (8,10). In this study, we evaluated lipid profile and CIMT in pediatric patients with SH.

## METHODS

The study included a patient group comprising 38 children diagnosed with SH and a control group comprising 38 healthy children with normal thyroid functions [serum TSF, free triiodothyronine (fT_3_), fT_4_] who presented to the Pediatric Endocrinology Department of Dicle University Faculty of Medicine, in Diyarbakır, Turkey, between April-August 2016. Normal ranges of our laboratory were as follows: TSH 0.27-4.2 mIU/L, fT_3_ 3.69-9.85 pmol/L, and fT_4_ 12-22.8 pmol/L. The diagnosis of SH was based on an elevated TSH level (4.2-20 mIU/L) and normal fT_4_ level measured in two morning fasting blood samples obtained at an interval of 2 to 6 weeks.

No subject in the study or control group had any sign or symptom of hypertension, liver and kidney dysfunction, lung disease, systemic infection, or any chronic disease. Patients with diabetes mellitus, obesity, and a history of drug use were excluded from the study. In both groups, no participant was using any medication during the study. In all subjects, the electrocardiogram (ECG) showed normal sinus rhythm and conventional transthoracic echocardiography findings were normal. Prior to the study, a written consent was obtained from the parents of each subject and an ethical approval was received from the local ethics committee.

Serum TSH, fT_3_, and fT_4_ levels were determined by using a Cobos e601 analyzer (Roche HITACHI Germany) with electrochemiluminescence immunoassay (ECLIA) method. Blood samples were collected by venipuncture in the morning after an overnight fast. Serum levels of total cholesterol (TC), high-density lipoprotein cholesterol (HDL-C), and triglycerides (TG) were determined by using a photometric method (Abbott diagnostics C16000 chemistry analyzer, Illinois, USA). Calculation of the value of low-density lipoprotein cholesterol (LDL-C) was performed using the Friedewald formula (11).

### Carotid Intima-Media Thickness Measurements

CIMT was determined by ultrasonographic images of the right carotid artery which were recorded with a 12 MHz linear array transducer (Vivid S5 Pro, GE, Horten, Norway). The patient was placed in the supine position with the neck slightly extended and the head rotated 45° to the opposite direction. The M-mode cursor was positioned 1.0 cm proximal to the right carotid artery bulb during end diastole. CIMT was accepted as the distance between the lumen-intima and the media-adventitia interfaces. The CIMT on the frozen frame of a suitable longitudinal image was manually measured off-line. The value of CIMT was determined based on the mean value of a minimum of three measurements. All the CIMT evaluations were performed by an experienced pediatric cardiologist blinded to the clinical and biochemical characteristics of the patients.

### Statistical Analysis

Data were analyzed using IBM SPSS 21.0 for Windows (SPSS Inc., Chicago, IL, USA). Quantitative variables were expressed as mean ± standard deviation (SD) and categorical variables were presented as count and percentage (%). Binary variables were compared by using independent samples t-test for normally distributed variables and by using Mann-Whitney U-test for non-normally distributed variables. Qualitative variables were compared by using chi-square test and the relationship among numerical variables was analyzed by using Pearson’s correlation coefficient. The hypotheses were two-tailed and a p-value of ≤0.05 was accepted statistically significant.

## RESULTS

The patient group included 38 children (16 male, 22 female) with SH and the control group included 38 healthy, euthyroid children (20 male, 18 female). Mean age was 8.1±3.6 (range, 3.5-15) years in the patient group and 8.9±2.4 (range, 4.5-15) years in the control group. No significant difference was found between the groups with regard to age, gender, body weight, and SD score (SDS) values for body mass indices. Table 1 presents the demographic profiles of the subjects in the study and control groups.

In the patient group, TSH level was significantly higher (p<0.001) and the fT_3_ and fT_4_ levels were similar compared to the control group. Total TC, LDL, TC/HDL, and LDL/HDL were also higher in the patient group compared to the control group (p=0.049, p=0.014, p=0.002, and p=0.003, respectively). In the patient group, 9 children were detected as having high lipid levels. Of these, 3 children had high TC levels (max. 225 mg/dL), 5-high TG levels (max. 275 mg/dL) and one patient had both high TG and TC levels. In the control group, 3 children had slightly high TG levels (max. 140 mg/dL). CIMT was significantly higher in the patient group compared to the control group (p=0.001). Table 2 presents the laboratory parameters and the CIMT values for both groups. There was no relationship between CIMT and lipid levels, and no correlations were detected between increasing lipid level and CIMT.

The patient group was further divided into two subgroups depending on serum TSH level: (I) patients with mildly elevated TSH (TSH=4.2±9.9 mIU/L) (n=33) and (II) patients with high TSH (TSH ≥10 mIU/L) (n=5). No significant difference was found between the two subgroups with regard to TC, LDL-C, HDL-C, TG, and CIMT (p=0.635, p=0.424, p=0.310, p=0.342, and p=0.610, respectively) (Table 3).

## DISCUSSION

The association between SH and lipid profile alteration has been reported in numerous studies (6,7,12). Moreover, TSH has been shown to induce the production of the hepatic 3-hydroxy-3-methyl-glutaryl coenzyme A (HMG CoA) reductase, which is a rate-limiting enzyme in cholesterol biosynthesis. Studies have also indicated that thyroid hormones may affect HDL metabolism by increasing the cholesteryl ester transfer protein activity and that they may also stimulate lipoprotein lipase (12).

The studies investigating the effect of SH on lipid profile alteration have reported contradictory findings. Although some studies found significant lipid profile changes in patients with SH (6,7,12,13), some others did not (14). In most of the studies that found significant lipid profile changes, increased TC, TG, and LDL levels, and decreased HDL levels have been reported (6,13,15,16,17,18).

Literature reviews indicate that studies investigating lipid profile changes in children with SH are few, as opposed to the large number of studies conducted in adults (7,19,20,21). A previous study evaluated both children and adults with SH and found no lipid abnormality in children with TSH levels <10 mIU/L and reported that the only abnormality was low HDL levels in children with TSH >10 mIU/L compared to controls. The study also reported low HDL levels in adults with TSH >10 mIU/L and, unlike in children, increased TC and LDL in adults (19). In our study on the other hand, no significant difference was found between the patients with TSH <10 mIU/L and those with TSH ≥10 mIU/L in terms of lipid profile. However, the small number of patients with TSH ≥10 mIU/L (n=5) included in this comparison was a disadvantage. Çatlı et al (20) evaluated 27 children with SH and found no significant difference in TG, HDL, and LDL levels in these patients compared to controls and suggested that SH is not associated with dyslipidemia in children with SH. Paoli-Valeri et al (21) evaluated 17 children with SH aged between 2-9 years and found significantly low HDL-C levels in these patients. Sert et al (8) compared obese children with and without nonalcoholic fatty liver disease (NAFLD) and found increased TC and LDL and decreased HDL levels in patients with NAFLD compared to children without NAFLD. Contrariwise, in our study, we evaluated non-obese children with SH and, to our knowledge, there has been no study reporting on increased TC and LDL in non-obese children with SH in the literature. However, there are reports of several studies similar to our study suggesting that SH causes no significant difference in HDL-C level but may increase the TC/HDL-C or LDL-C/HDL-C ratios (6).

A correlation between CIMT and cardiovascular disease has been frequently reported in epidemiological studies, indicating that increased CIMT is a reliable marker for subclinical atherosclerosis (22). However, although the frequency of hypertension and dyslipidemia is remarkably high in patients with SH, the association between SH and CIMT has been shown to be independent from these two conditions (8). In addition, another study showed that SH leads to increased risk of myocardial infarct and atherosclerosis, independent of serum cholesterol levels (23).

The correlation between SH and CIMT has mostly been reported in adult patients (8,24,25,26). To our knowledge, there are only two publications in the literature reporting an increased CIMT in children with SH (7,10). In one of these, Isik-Balci et al (11) evaluated 53 children with SH and reported that CIMT was significantly increased in children with SH compared to controls (0.48±0.04 mm vs. 0.43±0.03, respectively). The other study, which was conducted by Sert et al (8), found that CIMT was significantly increased in obese children with NAFLD compared to obese children without NAFLD and these authors have also reported that CIMT had a positive correlation with TSH. A meta-analysis investigating the correlation between SH and CIMT in adults concluded that CIMT was more prevalent in SH patients with TSH >10 mIU/L and suggested that CIMT was increased in patients with TSH <10 mIU/L as well, though slightly (8). Delitala et al (27) evaluated subclinical thyroid disorders (subclinical hypo- and hyperthyroidism) in 5,815 individuals aged between 14-102 years and reported that there was no association between these disorders and CIMT. However, the study had an important shortcoming since the TSH and fT_4_ measurements were performed only once; instead, these measurements should be repeated for a second time, since slightly increased TSH levels have been shown to return to normal in the subsequent measurement in almost 70% of the patients (28). To the best of our knowledge, the present study is the third study in the literature reporting a significantly increased CIMT in children with SH.

In conclusion, our study revealed increased TC, LDL-C, TC/HDL and LDL/HDL levels and a significant increase in CIMT in non-obese children with SH, findings which have been scarcely reported in the literature. Since dyslipidemia and increased CIMT are accepted as risk factors for cardiovascular diseases, L-thyroxine could be considered for the treatment of SH. Future studies with larger sample sizes and longer periods of follow-up are needed to further substantiate the importance of L-thyroxine treatment in SH patients to decrease the risk of cardiovascular diseases.

## Figures and Tables

**Table 1:**
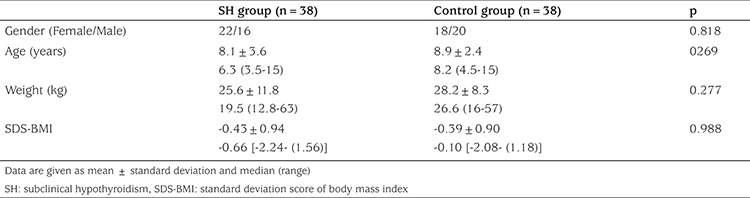
Demographic characteristics of the study and control groups

**Table 2:**
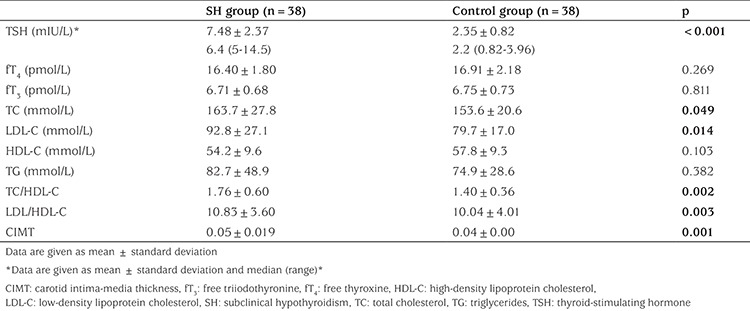
Biochemical characteristics and carotid intima-media thickness of the study groups

**Table 3:**
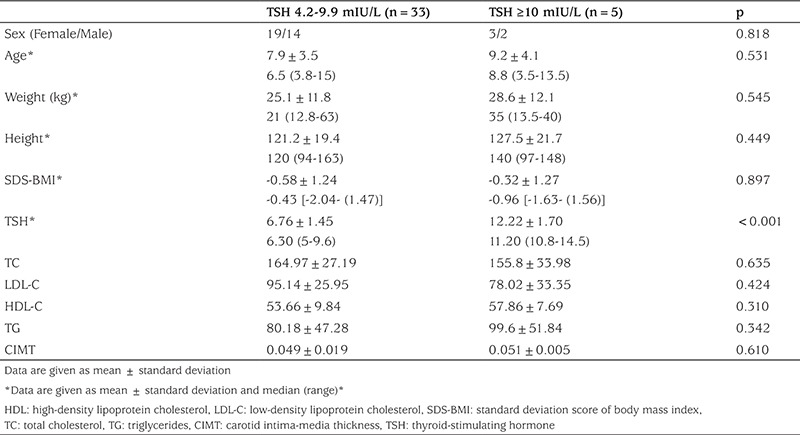
Demographic characteristics, laboratory parameters, and carotid intima-media thickness values of the patients with thyroid-stimulating hormone <10 mIU/L and thyroid-stimulating hormone ≥10 mIU/L
